# Steps/day translation of the moderate-to-vigorous physical activity guideline for children and adolescents

**DOI:** 10.1186/1479-5868-10-49

**Published:** 2013-04-21

**Authors:** Marc A Adams, William D Johnson, Catrine Tudor-Locke

**Affiliations:** 1 Exercise and Wellness Program, School of Nutrition and Health Promotion, Arizona State University, 500 N. Third Street, (Mail Code 3020), Phoenix, AZ, 85004, USA; 2 Biostatistics Core, Pennington Biomedical Research Center, Louisiana State University, 6400 Perkins Road, Baton Rouge, LA, 70808, USA; 3 Walking Behavior Laboratory, Pennington Biomedical Research Center, Louisiana State University, 6400 Perkins Road, Baton Rouge, LA, 70808, USA

**Keywords:** Pedometer, Exercise, Sensitivity and specificity, Translation, Centers for disease control and prevention, CDC, National health and nutrition examination survey, NHANES

## Abstract

**Background:**

An evidence-based steps/day translation of U.S. federal guidelines for youth to engage in ≥60 minutes/day of moderate-to-vigorous physical activity (MVPA) would help health researchers, practitioners, and lay professionals charged with increasing youth’s physical activity (PA). The purpose of this study was to determine the number of free-living steps/day (both raw and adjusted to a pedometer scale) that correctly classified children (6–11 years) and adolescents (12–17 years) as meeting the 60-minute MVPA guideline using the 2005–2006 National Health and Nutrition Examination Survey (NHANES) accelerometer data, and to evaluate the 12,000 steps/day recommendation recently adopted by the President’s Challenge Physical Activity and Fitness Awards Program.

**Methods:**

Analyses were conducted among children (n = 915) and adolescents (n = 1,302) in 2011 and 2012. Receiver Operating Characteristic (ROC) curve plots and classification statistics revealed candidate steps/day cut points that discriminated meeting/not meeting the MVPA threshold by age group, gender and different accelerometer activity cut points. The Evenson and two Freedson age-specific (3 and 4 METs) cut points were used to define minimum MVPA, and optimal steps/day were examined for raw steps and adjusted to a pedometer-scale to facilitate translation to lay populations.

**Results:**

For boys and girls (6–11 years) with ≥ 60 minutes/day of MVPA, a range of 11,500–13,500 uncensored steps/day for children was the optimal range that balanced classification errors. For adolescent boys and girls (12–17) with ≥60 minutes/day of MVPA, 11,500–14,000 uncensored steps/day was optimal. Translation to a pedometer-scaling reduced these minimum values by 2,500 step/day to 9,000 steps/day. Area under the curve was ≥84% in all analyses.

**Conclusions:**

No single study has definitively identified a precise and unyielding steps/day value for youth. Considering the other evidence to date, we propose a reasonable ‘rule of thumb’ value of ≥ 11,500 accelerometer-determined steps/day for both children and adolescents (and both genders), accepting that more is better. For practical applications, 9,000 steps/day appears to be a more pedometer-friendly value.

## Introduction

In 2008 the U.S. Department of Health and Human Services recommended that children (up to age 11 years) and adolescents (ages 12–17 years) accumulate ≥ 60 minutes/day of moderate-to-vigorous physical activity (MVPA) to prevent obesity, and benefit their physical fitness, bone health, metabolic and cardiovascular risk factors, and symptoms of depression and anxiety [1,2]. When measured objectively using accelerometer technology, the prevalence of meeting MVPA recommendations in the U.S. was 42% for children and < 8% for adolescents [3]. Encouraging children and adolescents to meet the MVPA guideline is a public health priority.

Body-worn motion sensors, such as pedometers and accelerometers, can be used by researchers and practitioners to objectively assess and promote daily physical activity. Pedometers are small and inexpensive devices that measure steps taken over time (or physical activity volume), usually expressed as steps/day [4]. However, a steps/day approximation of the aerobic component of the public health guideline, which is focused on MVPA, has not been possible because pedometers were not specifically designed to measure physical activity intensity or duration. In contrast, accelerometers offer a time-stamped technology that presents movement as a rate (i.e., activity counts/minute) that researchers can use to infer time spent at various intensities of physical activity. Certain accelerometers now also present time-stamped steps taken as a rate (i.e., steps/min). Accelerometers are generally much more expensive compared to typical research-grade pedometers and therefore are less feasible for parents, youth, and wide spread public health use. Simultaneously considering the accelerometer step and intensity outputs can potentially inform a pedometer-scaled steps/day value congruent with public health MVPA guidelines. However, relatively few data exist to inform a steps/day translation of the ≥60 minute/day MVPA guideline [5].

A recent review of studies conducted among children and adolescents from around the world concluded boys take on average 13,000–15,000 steps/day and girls take 11,000–12,000 steps/day [5]. Recently, Colley, Janssen, and Temblay analyzed MVPA and step count data collected with an Actical accelerometer from 1,613 Canadian children and adolescents (6–19 years of age) [6]. Applying a receiver operating curve (ROC) analysis, they determined that steps/day values equivalent to 60 minutes/day of MVPA ranged between 11,290 and 12,512 steps/day. They recommended that 12,000 steps/day was a “practical” single cut point useful for all age and gender groups. Although accelerometers are considered to be generally more sensitive to low force accelerations than pedometers, the degree to which the Actical accelerometer’s step data translates to more commonly used pedometers is unknown, and the researchers in this study did not attempt to adjust the data in any manner to make it more directly relevant to a pedometer-based scale. In the U.S., the Presidential Active Lifestyle Award (https://www.presidentschallenge.org/celebrate/active-lifestyle.shtml) adopted this 12,000 steps/day recommendation, acknowledging this Canadian research as the source.

The U.S. has its own source of nationally representative accelerometer data collected in the 2005–2006 National Health and Nutrition Examination Surveys (NHANES) using an ActiGraph accelerometer. In a concurrent laboratory comparison, the ActiGraph detected significantly more steps at slower walking speeds than the Actical [7]. A descriptive study of 2005–2006 NHANES data (expressed on a scale more consistent with pedometer output) reported that boys and girls ages 6–11 years averaged approximately 10,600 and 9,500 steps/day, respectively [8]. Adolescent boys and girls (defined in that paper as ages 12–19 years) averaged approximately 8,800 and 6,700 steps/day, respectively. No study to date has evaluated a steps/day translation of the MVPA guideline in this nationally representative sample of U.S. children and adolescents. Therefore, the purpose of this analysis of the NHANES accelerometer data was to 1) examine U.S. data to identify the number of free-living steps/day (both raw (uncensored) and adjusted to a pedometer scale) that more frequently correctly classified children (6–11 years) and adolescents (12–17 years, following the age definitions included in the federal public health recommendations) as meeting the ≥60 minute/day MVPA guideline and 2) evaluate the 12,000 steps/day recommendation established with Canadian data using a different accelerometer.

## Methods

The publically-accessible 2005–2006 NHANES accelerometer data were used for this analysis. The cross-sectional NHANES sampled non-institutionalized U.S. youth and adults using a multi-stage probability approach to oversample adolescents, non-Hispanic Blacks, and Hispanics [9]. Trained interviewers visited selected households and surveyed eligible participants. Ambulatory individuals ages 6 years or older who subsequently attended a mobile health examination were invited to participate in an accelerometer sub-study. Standardized survey instruments, protocols and datasets are available online (http://www.cdc.gov/nchs/nhanes.htm). The Institutional Review Board at the U.S. National Center for Health Statistics (NCHS) approved all NHANES protocols. Adults and adolescents provided written consent, and assent was obtained from children under 16 years of age along with written informed consent from a parent. Boys and girls 6 to 17 years of age (reflecting specified ages in the 2008 physical activity guidelines [1]) who participated in the accelerometer sub-study of NHANES were included in these analyses.

### Measures

#### Demographic measures

Trained NHANES interviewers conducted the comprehensive interview that asked participants or a parent for the child’s / adolescent’s age, gender, and ethnicity.

#### Accelerometer measures

Participants in the accelerometer sub-study wore a uniaxial ActiGraph 7164 accelerometer (Walton Beach, FL) on the right hip using an elastic belt during all waking hours (instructed to remove for swimming or bathing) for 7 days. The accelerometer was initialized to simultaneously measure both activity counts and steps taken in 1-minute epochs. ActiGraph accelerometers have produced reliable and valid estimates of youth MVPA in laboratory and free-living conditions [10,11].

### Data treatment

#### Accelerometer activity counts

Participants’ accelerometer records were available from the NHANES Physical Activity Monitor (PAM) dataset. The PAM dataset was scored using SAS code provided by National Cancer Institute available at: http://riskfactor.cancer.gov/tools/nhanes_pam. For scoring accelerometer-derived MVPA, a ‘valid day’ was defined as one in which the participant had ≥ 10 valid hours of wear time. Invalid hours were subtracted from 24 hours to calculate valid hours. An invalid hour consisted of at least 60 consecutive minutes of zero-value activity counts with allowance of 1 to 2 minutes of between 1 and 100 activity counts. On valid days, each valid minute was scored as meeting or not meeting a criterion activity count associated with at least moderate intensity. These activity counts were based on age-specific Freedson cut points for youth, using both 3 METs and 4 METs to define the minimum threshold of moderate intensity, and Evenson cut points [12-14]. The original calibration studies that derived Freedson and Evenson cut points used the ActiGraph 7164, the same instrument as used in NHANES. A recent review reported a lack of consensus on the MET level that defines a minimum threshold of moderate intensity activity and scoring protocols for accelerometer data [15]. Current national public health guidelines specify a minimum of 3 METs, [2] but some authors have argued for and used a minimum of 4 METs for youth populations based on recent calibration studies [3,13]. Furthermore, a recent study reported that Evenson cut points (~3 METs) [14] out-performed the Freedson 4 METs for children under 10 years of age [16]. Therefore, faced with this cut point conundrum, we examined the data using the three different MVPA cut points together with commonly used scoring decisions. Table 1 presents the specific activity count cut points used in this analysis to define MVPA by minimal MET level and age.

**Table 1:** Age-adjusted Freedson and Evenson accelerometer activity count/min thresholds for children and adolescents

	**Activity count/min thresholds**		
**Age (years)**	**Freedson (3 METs)**	**Freedson (4 METs)**	**Evenson**
6	614	1400	2296
7	705	1515	2296
8	802	1638	2296
9	906	1770	2296
10	1017	1910	2296
11	1135	2059	2296
12	1262	2220	2296
13	1399	2393	2296
14	1546	2580	2296
15	1706	2781	2296
16	1879	3000	2296
17	2068	3239	2296

#### Accelerometer-determined steps/day

Steps were estimated by within-instrument processing of the number of cycles in the accelerometer signal or “cycle counts” [17]. The ActiGraph step feature has been validated against directly observed steps with normal- and over-weight youth [18,19]. However, because the ActiGraph 7164 accelerometer is more sensitive to low force accelerations compared to research-grade pedometers, and also because one of the objectives was to provide a readily translatable step/day translation of time in MVPA, its step output was adjusted to make it more comparable to research-grade pedometers (which are more likely to be used in public health and clinical applications) by censoring those steps taken below 500 activity counts/min. This censoring approach was based on previous empirical and sensitivity analyses conducted with NHANES adults and youth data [8,20].

Participants with at least one valid day were included in the present analyses. This criteria is similar to other analyses using NHANES accelerometer data [8,21]. A previous examination found that youth with four or more valid days took approximately 1,700 more steps/ day than those with fewer valid days, suggesting that excluding participants resulted in a sampling bias that inflated average values [8]. For all valid days the number of 1-minute epochs where activity counts reached or exceeded the moderate-intensity threshold were summed and divided by the number of valid days to calculate the average minutes/day spent in MVPA. Children and adolescents who averaged ≥60 minutes/day of MVPA were classified as having met the federal MVPA guideline [1]. Average daily uncensored and censored steps were computed by summing steps accumulated for each day across valid days and dividing by the number of valid days.

#### Statistical analysis

Analyses were conducted in 2011 and 2012 using SAS version 9.2 (SAS Institute, Cary, NC) to invoke procedures for survey data and account for NHANES’ complex sampling design and sample weights. Results were summarized as means, frequencies and proportions, as appropriate. A ROC analysis was used to investigate incremental increases in plausible steps/day cut points that could be employed to discriminate attainment of ≥ 60 minutes of accelerometer-derived time in MVPA. Results for both uncensored and censored steps/day are reported separately for children (6–11 years) and adolescents (12–17 years) by gender, and two definitions of moderate intensity (i.e., Freedson 3 and 4 METs), and Evenson cut points.

ROC plot figures and (mis)classification statistics were determined in 500 step/day increments ranging from 6,500 to 14,500 steps/day. Classification indices included: sensitivity (true positive), specificity (true negative), false positive, false negative, positive predictive value (PPV), negative predictive value (NPV), accuracy, and area under the curve (AUC). Positive predictive values provide an estimated probability of a true positive result given the observation of a positive test, or in the current context the probability of truly attaining ≥ 60 MVPA minutes for an individual given a positive indication that they accumulated a specific number of steps/day or more (e.g., 10,500). Negative predictive values provide an estimated probability of a true negative given a negative test is observed. Or for the current context, the probability of an individual truly not accumulating ≥ 60 MVPA minutes when he/she has attained fewer than a specific number of steps/day. Accuracy is defined as the sum of the number of true positives and true negatives divided by the number of individuals tested. AUC is a global test that indicates the probability that a random individual from the positive outcome group (i.e., attained ≥ 60 MVPA minutes/day) had a higher steps/day value than a random individual from the negative outcome group (i.e., attained < 60 minutes/day) [22]. AUC values were judged based on published standards: ≥0.90 were considered excellent, 0.80–0.89 good, 0.70–0.79 fair, and <0.70 poor [23]. Selection of optimal steps/day was derived from balancing false positives (i.e., inactive youth misclassified as meeting physical activity guideline) and false negatives (i.e., youth meeting guideline misclassified as inactive) [24]. We also evaluated the Canadian’s Actical-derived 12,000 steps/day threshold for false positives and negatives across subgroups.

## Results

The sample consisted of 2,468 youth aged 6–17 years of age. Of the total sample, 2,217 (89.8%; 915 children 6–11 years and 1,302 adolescents 12–17 years) had at least one valid day of monitoring; 251 youth were excluded from analyses because they provided <10 hours of valid data on any single day. Table 2 presents weighted descriptive statistics for the analytic sample. In general, children were more physically active than adolescents, and boys (both children and adolescents) were more active than girls (i.e., greater average steps/day and durations of MVPA/day obtained using comparable MET thresholds). Average accelerometer activity counts/min, which are not affected by any of the cut point intensity definitions applied, confirmed this pattern of lower activity for girls and adolescents.

**Table 2:** **Weighted descriptive characteristics for children and adolescents by gender, NHANES 2005-2006**^**1**^

	**Children (6–11 years)**				**Adolescents (12–17 years)**			
	**Boys (n = 442)**		**Girls (n = 473)**		**Boys (n = 646)**		**Girls (n = 656)**	
	**Mean**	**SE**	**Mean**	**SE**	**Mean**	**SE**	**Mean**	**SE**
Age (years)	8.62	0.11	8.53	0.08	14.40	0.11	14.52	0.11
Ethnicity (%)								
Non-hispanic white	57.70	4.94	55.22	4.11	62.95	4.79	60.56	3.30
Non-hispanic black	14.07	2.89	14.62	2.20	14.91	3.18	15.07	2.68
Mexican American	14.68	1.72	14.29	1.64	12.23	1.41	11.61	1.58
Other	13.55	2.92	15.88	3.11	9.91	1.60	12.75	2.46
MVPA minutes/day (FR 3 METs)	191.79	5.76	169.99	3.84	75.05	3.06	47.80	2.22
MVPA minutes/day (FR 4 METs)	94.14	3.11	76.53	2.24	35.37	1.60	18.94	1.11
MVPA minutes/day (EV)	58.28	1.89	44.76	1.68	43.50	1.40	25.21	1.13
% ≥60 min of MVPA (FR 3 METs)	95.03	2.31	98.03	0.87	57.31	3.40	27.39	2.86
% ≥60 min of MVPA (FR 4 METs)	74.75	2.66	61.46	3.67	14.31	1.68	2.89	0.84
% ≥60 min of MVPA (EV)	45.85	2.39	22.01	2.90	22.05	2.34	6.66	1.84
Steps/day (uncensored)	13,088	271	12,313	274	11,489	235	9,449	206
Steps/day (censored)	10,530	242	9,544	246	8,922	221	6,885	184
Activity counts/min	649.47	15.67	589.01	10.85	476.15	11.99	353.18	10.31

^1^Values and standard errors were estimated after accounting for NHANES complex survey design and sample weights. Sample sizes refer to unweighted values. All values were derived from participants with 1 to 7 valid days of accelerometer data. Of the 2,217, 197 youth had only 1 valid day and 1,643 youth had 4 or more valid days.

Table 3 provides a summary of ROC results (detailed ROC results made available in the Additional file 1) including optimal steps/day values for censored and uncensored data by MVPA cut point (i. e., Freedson 3, Freedson 4, Evenson) age group, and gender. Table 3 also shows AUC values, classification accuracy, false positive and false negatives for children and adolescents, respectively, by optimal steps/day values and 12,000 steps/day. Additional file 1 tables show the ranges of associated classification statistics for 6,500 to 14,500 steps/day cut points for children and adolescents based on censored (Additional file 1: Tables S1a-S6b) and uncensored steps (Additional file 1: Tables S7a-S12b) by gender and MVPA cut point.

**Table 3:** **Optimal steps/day from ROC analysis and Colley et al. thresholds**^**a **^**for censored and uncensored steps by activity cut point for children and adolescents**

		**Children**				
	**Freedson 3 METS**		**Freedson 4 METS**		**Evenson**	
	**Optimal**	**Colley**^**a**^	**Optimal**	**Colley**^**a**^	**Optimal**	**Colley**^**a**^
**Censored**						
Boys 6–11 yrs	6,500	12,000	9,500	12,000	10,500	12,000
FP/FN^b^	(0%/6%)	(0%/68%)	(17%/21%)	(0%/59%)	(19%/20%)	(5%/39%)
Accuracy	.94	.35	.80	.56	.81	.80
AUC	.99 (.98–1.0)		.91 (.88–.94)		.90 (.87–.93)	
Girls 6–11 yrs	6,500	12,000	9,000	12,000	10,500	12,000
FP/FN^b^	(0%/15%)	(0%/83%)	(15%/21%)	(1%/74%)	(21%/15%)	(6%/42%)
Accuracy	.85	.19	.81	.55	.80	.86
AUC	.88 (.85–.91)		.90 (.88–.93)		.88 (.85–.92)	
**Uncensored**						
Boys 6–11 yrs	8,500	12,000	12,000	12,000	13,000	12,000
FP/FN^b^	(9%/6%)	(0%/33%)	(20%/21%)	(20%/21%)	(26%/21%)	(43%/9%)
Accuracy	.94	.68	.79	.79	.76	.72
AUC	.99 (.97–1.0)		.88 (.85–.92)		.86 (.83–.90)	
Girls 6–11 yrs	8,500	12,000	11,500	12,000	13,500	12,000
FP/FN^b^	(0%/9%)	(0%/48%)	(24%/19%)	(17%/27%)	(22%/27%)	(42%/11%)
Accuracy	.91	.53	.79	.77	.77	.65
AUC	.97 (.95–1.0)		.87 (.84–.90)		.84 (.80–.89)	
		**Adolescents**				
	**Freedson 3 METS**		**Freedson 4 METS**		**Evenson**	
	**Optimal**	**Colley**^**a**^	**Optimal**	**Colley**^**a**^	**Optimal**	**Colley**^**a**^
**Censored**						
Boys 12–17 yrs	8,500	12,000	11,500	12,000	10,500	12,000
FP/FN^b^	(21%/21%)	(1%/68%)	(11%/14%)	(8%/22%)	(15%/21%)	(3%/34%)
Accuracy	.79	.61	.88	.90	.83	.90
AUC	.99 (.97–1.0)		.93 (.91–.96)		.93 (.91–.95)	
Girls 12–17 yrs	7,500	12,000	9,000	12,000	9,500	12,000
FP/FN^b^	(23%/16%)	(0%/82%)	(18%/19%)	(4%/52%)	(12%/13%)	(2%/41%)
Accuracy	.79	.78	.82	.95	.88	.95
AUC	.88 (.86–.91)		.87 (.78–.96)		.93 (.88–.98)	
**Uncensored**						
Boys 12–17 yrs	11,000	12,000	14,000	12,000	13,000	12,000
FP/FN^b^	(28%/24%)	(16%/38%)	(15%/17%)	(34%/6%)	(21%/19%)	(29%/13%)
Accuracy	.74	.72	.84	.70	.80	.75
AUC	.84 (.82–.87)		.91 (.88–.94)		.89 (.87–.92)	
Girls 12–17 yrs	10,500	12,000	11,500	12,000	12,000	12,000
FP/FN^b^	(24%/24%)	(9%/52%)	(23%/19%)	(19%/24%)	(17%/18%)	(17%/18%)
Accuracy	.76	.79	.77	.81	.83	.83
AUC	.85 (.81–.88)		.84 (.73–.95)		.90 (.83–.96)	

^a^ Colley et al. thresholds are 12,000 steps/day across gender and age groups.

^b^FP/FN is false positives and false negatives.

Accuracy = (true positives + true negatives)/(true positives + true negatives + false positives + false negatives).

AUC = area under the curve.

### Results for children 6–11 years old (Freedson 3 METs)

Over 95% of children (both boys and girls) attained ≥ 60 minutes/day of MVPA using the Freedson 3 MET threshold for MVPA (Table 2). Boys averaged 3.2 hours/day and girls averaged 2.8 hours/day of 3 MET-defined MVPA.

#### Censored steps/day

For both boys and girls, 6,500 steps/day produced the optimal balance between false positives and negatives for attainment of ≥ 60 minutes/day of MVPA across all candidate cut points (Table 3; Additional file 1: Table S1a-b). Accuracy was 85%–94% for 6,500 steps. For 12,000 steps/day, 100% sensitivity (0% false positives) was observed for both boys and girls with 68% and 83% false negatives, respectively. Accuracy was 35% for boys and 19% for girls. AUC was excellent at 99% for boys and good (88%) for girls.

#### Uncensored steps/day

Approximately 8,500 steps/day produced the best balance across all candidate step values for meeting the MVPA guideline for both boys and girls (Table 3; Additional file 1: Table S7a-b). Accuracy was 94% for boys and 91% for girls. For 12,000 steps/day, 100% sensitivity was observed for both boys and girls with 33% and 48% false negatives, respectively (Table 3). Accuracy was 68% for boys and 53% for girls. AUC was excellent at ≥97% for both genders across analyses.

### Results for children 6–11 years old (Freedson 4 METs)

Compared to the 3 MET threshold, fewer children (approximately 75% of boys and 61% of girls) attained ≥60 minutes/day of MVPA using a 4 MET threshold (Table 2). Boys and girls averaged 1.6 and 1.3 hours/day of 4 MET-defined MVPA, respectively.

#### Censored steps/day

For boys and girls, 9,500 and 9,000 steps/day revealed the optimal balance between false positives and negatives, respectively (Table 3; Additional file 1: Table S2a-b). Accuracy was 80% for boys and 81% for girls. For 12,000 steps/day, 100% sensitivity was observed for boys and 99% for girls with 59% and 74% false negatives, respectively (Table 3). Accuracy was 56% for boys and 55% for girls. AUC was excellent at ≥90% for both genders.

#### Uncensored steps/day

For boys and girls, 12,000 and 11,500 steps/day were associated with the optimal balance between false positives and negatives, respectively (Table 3; Additional file 1: Table S8a-b). Accuracy was 79% for both genders. For 12,000 steps/day, 80% sensitivity was observed for boys and 83% for girls with 21% and 27% false negatives, respectively (Table 3). Accuracy was 79% for boys and 77% for girls. AUC was good at ≥87% for both genders.

### Results for children 6–11 years old (Evenson)

Approximately 46% of boys and 22% of girls attained ≥ 60 minutes/day of MVPA using the Evenson threshold for MVPA (Table 2). Boys averaged 58.3 minutes/day and girls averaged 44.8 minutes of MVPA.

#### Censored steps/day

For both gender groups, 10,500 steps/day was associated with the optimal balance between false positives and negatives (Table 3; Additional file 1: Table S3a-b). Accuracy was 81% for boys and 80% for girls. For 12,000 steps/day, 95% sensitivity was observed for boys and 94% for girls with 39% and 42% false negatives, respectively (Table 3). Accuracy was 80% for boys and 86% for girls. AUC was good-to-excellent at ≥88% for both genders.

#### Uncensored steps/day

For boys 13,000 and for girls 13,500 steps/day was associated with the optimal balance between false positives and negatives (Table 3; Additional file 1: Table S9a-b). Accuracy was 76% for boys and 77% for girls. For 12,000 steps/day, 57% sensitivity was observed for boys and 58% for girls with 9% and 11% false negatives, respectively. Accuracy was 72% for boys and 65% for girls. AUC was good at ≥84% for both genders.

### Results for adolescents 12–17 years old (Freedson 3 METs)

Table 2 shows that 57% of adolescent boys and 27% of girls attained ≥ 60 minutes/day of MVPA defined using 3 METs. Adolescent boys averaged 1.3 hours/day of 3 MET-defined MVPA and girls averaged < 60 minutes/day.

#### Censored steps/day

For boys 8,500 and for girls 7,500 steps/day was associated with the optimal balance between false positives and negatives (Table 3; Additional file 1: Table S4a-b). For both genders, accuracy was 79%. For 12,000 steps/day, 99% sensitivity was observed for boys and 100% for girls with 68% and 82% false negatives, respectively (Table 3). Accuracy was 61% for boys and 78% for girls. AUC for all analyses was good for girls (88%) and excellent for boys at 99%.

#### Uncensored steps/day

For boys, 11,000 and for girls 10,500 steps/day was associated with the optimal balance between false positives and negatives (Table 3; Additional file 1: Table S10a-b). Accuracy was 74% for boys and 76% for girls. For 12,000 steps/day, 84% sensitivity was observed for boys and 91% for girls with 38% and 52% false negatives, respectively (Table 3). Accuracy was 72% for boys and 79% for girls. AUC was good at ≥84% for both genders.

### Results for adolescents 12–17 years old (Freedson 4 METs)

The majority of adolescent boys and girls did not attain ≥ 60 minutes/day of MVPA defined using Freedson 4 METs (Table 2). Notably, fewer than 15% of adolescent boys and fewer than 3% of adolescent girls achieved the 4 MET-defined recommended duration of MVPA.

#### Censored steps/day

For boys 11,500 and for girls 9,000 steps/day was associated with the optimal balance between false positives and negatives (Table 3; Additional file 1: Table S5a-b). Accuracy was 88% for boys and 82% for girls. For 12,000 steps/day, 92% sensitivity was observed for boys and 96% for girls with 22% and 52% false negatives, respectively (Table 3). Accuracy was 90% for boys and 95% for girls. AUC was excellent at 93% for adolescent boys and good at 87% for adolescent girls.

#### Uncensored steps/day

For boys 14,000 and for girls 11,500 steps/day was associated with the optimal balance between false positives and negatives (Table 3; Additional file 1: Table S11a-b). Accuracy was 84% for boys and 77% for girls. For 12,000 steps/day, 66% sensitivity was observed for boys and 81% for girls with 6% and 24% false negatives, respectively (Table 3). Accuracy was 70% for boys and 81% for girls. AUC was excellent at 91% for boys and good at 84% for girls.

### Results for adolescents 12–17 years old (Evenson)

Similar to Freedson 4 METs, the majority of adolescent boys and girls did not attain ≥ 60 minutes/day of MVPA defined using Evenson cut points (Table 2). However, compared to Freedson 4 cut points, a greater number of adolescent boys (22%) and adolescent girls (7%) achieved the recommended duration of MVPA. This was because the absolute count value (2,296 activity counts) associated with MVPA is lower for Evenson than Freedson 4 METs for 13 to 17 year olds (see Table 1). Adolescent boys averaged over 43-minutes/day of MVPA and adolescent girls averaged 25 minutes/day.

#### Censored steps/day

For boys 10,500 and for girls 9,500 steps/day was associated with the optimal balance between false positives and negatives (Table 3; Additional file 1: Table S6a-b). Accuracy was 83% for boys and 88% for girls. For 12,000 steps/day, 97% sensitivity was observed for boys and 98% for girls with 34% and 41% false negatives, respectively (Table 3). Accuracy was 90% for boys and 95% for girls. AUC was excellent at 93% for both genders.

#### Uncensored steps/day

For boys 13,000 and for girls 12,000 steps/day was associated with the optimal balance between false positives and negatives (Table 3; Additional file 1: Table S12a-b). For both genders, accuracy was 80% for boys and 83% for girls. For 12,000 steps/day, 71% sensitivity was observed for boys and 83% for girls with 13% and 18% false negatives, respectively. Accuracy was 75% for boys and 83% for girls (same as optimal). AUC was good to excellent at 89% and 90%.

## Discussion

A persistent question in the physical activity literature has been “How many steps/day are enough?” [5,24,25]. Although many studies have reported the normative or average daily steps accumulated by children and adolescents, few studies have estimated how many steps/day are needed for youth to truly meet the aerobic component of the public health guidelines as currently prescribed. This analysis of accelerometer step and activity count data collected concurrently in a nationally representative U.S. sample demonstrated that for those 6–11 years old who attained ≥ 60 minutes/day of MVPA on average, the optimal steps/day thresholds ranged from ≥ 6,500 to ≥10,500 steps/day (censored) and from ≥8,500 to ≥13,500 steps/day (uncensored), depending on the activity count cut point used to define moderate intensity. For adolescents (i.e., 12–17 years of age) who attained ≥60 minutes/day of MVPA on average, optimal steps/day thresholds ranged from ≥7,500 to 11,500 steps/day (censored) and from ≥ 10,500 to 14,000 steps/day (uncensored), depending on the activity count cut point that defined moderate intensity.

Colley, Janssen and Tremblay recently recommended 12,000 steps/day based on objectively monitored data collected from Canadian children using an Actical accelerometer and unadjusted to a pedometer-based scale [6]. They reported that a range between 11,290 and 12,512 steps/day best predicted recommended time in MVPA across age and gender groups, but concluded 12,000 steps/day as a single practical value across subgroups. Based on this recommendation, we explored how the 12,000 steps/day value would function in the NHANES dataset collected using a different accelerometer, in a different population, and across ActiGraph-derived activity count cut points with censored and uncensored steps (uncensored steps would be the most direct comparison to Actical). For boys and girls 6–11, the maximum false positive value was 6% for censored steps and 43% for uncensored steps across the three types of MVPA cut points used to define ≥60-minutes of MVPA. However, the maximum false negative value was 83% for censored steps and 48% for uncensored steps. Compared with the identified optimal threshold, the higher false negative values observed with the Colley et al. 12,000 steps/day threshold resulted in fewer children 6–11 correctly classified as meeting the guideline across the majority of comparisons. For adolescents, the maximum false positive value was 8% for censored and 34% for uncensored steps. Classification accuracies were more comparable between the identified optimal value and the Colley et al. steps/day values, even with higher false negative values observed for 12,000 steps/day. A 12,000 steps/day recommendation is higher than those steps/day values observed in the present analysis across different activity cut points for censored steps/day, thereby producing fewer false positives, but within the range observed for uncensored steps/day. This suggests that 12,000 steps/day works reasonably well for accelerometer-determined estimates of steps/day for Freedson 4 METs and Evenson cut points, but may be too high relative to Freedson 3 METs and less sensitive pedometer estimates.

The optimal steps/day threshold in the present analysis depended on gender and age group, the equation used to estimate average MVPA minutes/day, and whether or not accelerometer-determined steps were adjusted downwards to reflect less sensitive but commonly used pedometers. Of these factors, differences in steps/day by gender and age group were expected, but other differences across the various examinations of accelerometer data were difficult to ignore. Steps/day (censored or uncensored) values across most of the activity count cut point definitions were higher for adolescents than children. The ongoing (and apparently unavoidable) activity cut point conundrum with ActiGraph accelerometers and the 3 MET versus 4 MET debate to define minimum level of moderate activity produced two unfortunate additional layers of confusion. The Freedson 3 cut point suggested that >95% of children 6–11 attained 60 min/day or more of MVPA on average. This estimate is extremely high and was a concern, so the results from using Freedson 3 METS should be interpreted cautiously in the analyses and discussion even though the 3 MET threshold is the minimum used in federal physical activity guidelines. A recent study by Trost et al. compared the Freedson 4 METS to Evenson cut points in a field-based study with the ActiGraph GT1M accelerometer, and while both produced similar predictive accuracy (AUC) compared to indirect calorimetery, the Evenson cut points were less likely to overestimate MVPA for children under 10 years of age. However, as of this date there is no consensus among researchers regarding the ideal accelerometer calibration study or decision rules for scoring accelerometer data for youth [15]. On the face of the findings herein, the percent of the sample meeting MVPA guidelines and steps/day associated with the Evenson and Freedson 4 MET definitions in children were intuitively more acceptable than the Freedson 3 MET definition.

An optimal steps/day threshold also depended on whether one used uncensored ActiGraph steps (raw steps) or adjusted steps downwards (censored steps/day approach) to reflect a less sensitive but commonly used research-grade pedometers. The validity of the ActiGraph 7164 for counting steps compared to direct observation is excellent compared to newer accelerometer models (e.g. GT3X+) and many research-grade pedometers [19,26]. However, research-grade pedometer use may be more practical for the lay public and health practitioners, so censored steps/day allows these groups to integrate accelerometer results with pedometer-based literature and application [8,20,27,28]. Therefore, we examined NHANES data using the uncensored and censored approaches in an effort to be most useful to both the research and practice communities. The censoring approach used in our study adjusts data for each individual by censoring steps taken when activity level was less than 500 activity counts. However, our results suggest that, compared to uncensored steps/day value observed in the current analyses, the censored data produced (on average) ~2,500 fewer steps/day consistently across age and gender groups for estimating ≥60 MVPA minutes/day (ignoring the Freedson 3 METS). Although this is an average value, the simple subtraction of 2,500 steps from uncensored accelerometer steps/day guideline values when using a research grade pedometer is a crude but possibly useful conversion rule for health and wellness practitioners.

A recent review focused on “How many steps/day are enough?” assembled relevant studies conducted among children and adolescents from around the world [5]. It concluded from limited evidence to date that primary/elementary school children should be directed to take at least 13,000–15,000 steps/day (boys) and 11,000–12,000 steps/day (girls). The review’s step/day values were much higher than those obtained in the present study for children using the 3 MET definition for both censored (i.e., ≥ 6,500) and uncensored (ie. ≥8,500) steps/day. These review values were also generally higher than those observed with the Freedson 4 MET and Evenson definitions for boys and girls 6–11 for both censored and uncensored steps/day. Importantly, values reported in the review article were based on a limited number of studies employing different types of objective physical activity monitors.

The review also concluded that adolescents should be urged to take at least 10,000–11,700 steps/day. This range was based on a single study of adolescents that used the same type of accelerometer as used in NHANES to report a MVPA-to-steps/day translation. The study was conducted with free-living U.S. adolescents, who were primarily overweight girls, and reported that approximately 10,000 to 11,700 uncensored steps/day accurately classified adolescents as meeting the ≥60 minutes/day MVPA guideline using the Freedson 3 and 4 MET definitions, respectively [29]. The current analysis found that 11,500 uncensored steps/day was predictive of meeting the physical activity guideline in adolescent girls using the Freedson 4 METS definition, so these two results are in general agreement.

Given the numerous considerations, and recognizing the need to provide practical values to serve research and practice uses, a range of 11,500–13,500 uncensored steps/day for children and 11,500–14,000 uncensored steps/day for adolescents seems appropriate. A pedometer-friendly adaptation reduces these minimum values to 9,000 steps/day (a 2,500 step/day difference). Similar analyses of NHANES adults suggested that 7000–8000 pedometer-scaled steps/day were indicative of recommended amounts of MVPA [30]. These values represent the minimum number of steps/day, as current physical activity guidelines urge children and adolescents to attain even greater than 60 minutes of MVPA daily. Increasing steps/day recommendations to higher thresholds has the effect of minimizing false positives at the cost of accepting more false negatives, which may be an acceptable tradeoff given the obesity epidemic in the U.S. [31].

The U.S. President’s Challenge Physical Activity and Fitness Awards Program (PCPAFAP) recommended, until relatively recently, that youth between 6 and 17 years of age accumulate at least 13,000 steps/day for boys and 11,000 steps/day for girls [32]. These values can be traced to a single study that used pedometers by Vincent and Pangrazi among children 6 to 12 years old from two southwestern U.S. elementary schools from the same school district [33,5]. In August 2012, the PCPAFAP adjusted their award criteria to 12,000 steps/day for all youth based on surveillance data collected Canadian Health Measures Survey (CHMS) for 6–19 year olds using a different accelerometer (i.e., Actical) than that used in the NHANES [6]. The Canadian researchers concluded that 12,000 steps/day was an appropriate indicator of having achieved ≥ 60 minutes/day of MVPA. This value is within the range of uncensored values determined in the present analysis. However, there are a number of differences between CHMS and NHANES studies. The studies differed in the type of accelerometer used, activity cut points and MET values used to define MVPA (Actical is not plagued to the same extent by the cut point conundrum, perhaps in part because less research has been conducted with it at this time), unit of analysis, age/gender subgroups, analytical approach, and population. The present study may be more generalizable for several reasons. It was based on: a large and representative sample of U.S. children and adolescents restricted to the age ranges used by the guideline, a widely used accelerometer, the most commonly used and newly recommended ActiGraph activity count cut points, a state-of-the-art analytical approach, and also attempted to provide a steps/day value that would be congruent with less sensitive but commonly used commercial research-grade pedometers, appropriate for public health recommendations and applications.

A number of methodological strengths and limitations must be acknowledged. There is always a tradeoff between false positives and negatives when deciding how to define cut points. We chose to balance misclassification errors. Balancing the two types of errors is not always the ideal approach [34]. Others may select cut points based by minimizing false positives or negatives. Our Additional file 1 tables provide researchers and practitioners a range of steps/day values that can be used for such purposes. A prior sensitivity analysis of NHANES youth data examined a range of candidate values for censoring ActiGraph steps to reflect pedometer-determined steps [8]. The 500 activity counts/minute value produced steps/day estimates that were in line with expected values obtained from pedometers, however, we acknowledge that an exact conversion factor for translating accelerometer-determined steps/day to pedometer determined steps/day remains elusive and is likely to vary depending on the type and model of accelerometer used. However, it is widely accepted that accelerometers will record more steps than pedometers, so regardless the exact value, some adjustment will be needed to make the output of one instrument comparable to the other. Other limitations include questions about the stability of single day step/day estimate versus multiple days, fewer adolescent girls meeting the guideline defined using 4 METs, and almost all children meeting the guideline defined using 3 METs. The low PPV for adolescent girls at 4 METS (see Additional file 1) is concerning, and results for that analysis need to be interpreted with caution. This issue occurred because very few adolescent girls attained the guideline at 4 METs (3%), producing a restricted distribution compared to the other age, gender and cut point groupings. Physical activity guidelines include recommendations for aerobic as well as bone and muscle strengthening activities, with the recommendation that a majority of the 60 minutes of MVPA come from the aerobic component. It is possible that some activities may not be ambulatory and not register as steps taken. Frequency of meeting the aerobic component of the guideline was not considered, but youth are expected to attain ≥ 60-minute/day of MVPA every day, so these results should be applicable on a daily basis. Finally, the current study estimated the number of steps/day in the context of meeting MVPA guidelines. Some health professionals or interventionists may prioritize other indicators (e.g. step cadence) [35] or health related outcomes, such as overweight status [36].

It is apparent that there is no simple answer to the question regarding a step-based translation of recommended time in MVPA for children and adolescents. As additional studies are conducted and the body of evidence expands, it may be possible to further refine a meaningful range of steps/day that is generally congruent with the intensity and duration based physical activity guidelines. Any conclusion will undoubtedly have to be tolerated as a “rule of thumb” rather than a precise number. For now, and based on these data, 11,500 steps/day appears to be the most reasonable number applied to uncensored ActiGraph outputs for both children and adolescents. This value is not that different from the 12,000 uncensored steps/day recommendation based on the Actical. For practical applications however, 9,000 steps/day obtained from the pedometer-scaled analysis in the current study is a more pedometer-friendly value. Future studies with concurrently worn accelerometers and pedometers may produce a more refined MVPA translation of pedometer-determined steps/day.

## Competing interests

The authors declare that they have no competing interests.

## Authors’ contributions

MA conceptualized the paper and was the primary data analyst and writer of the paper. CTL and WJ contributed to analyses, writing, editing and approved the final manuscript. All authors read and approved the final manuscript.

## Supplementary Material

Additional file 1 Detailed ROC results for censored and uncensored steps/day by MVPA cut point (i. e, Freedson 3, Freedson 4, Evenson), age group, and gender. Click here for file
