# Supplementary material for: Steps/day translation of the moderate-to-vigorous physical activity guideline for children and adolescents
Source: Int J Behav Nutr Phys Act. 2013 Apr 21;10:49. doi: 10.1186/1479-5868-10-49 (PMC3639120; doi:10.1186/1479-5868-10-49)
Supplement: Additional file 1 — Detailed ROC results for censored and uncensored steps/day by MVPA cut point (i. e, Freedson 3, Freedson 4, Evenson), age group, and gender. [file 1479-5868-10-49-S1.doc]

| Additional file 1: Table S1a. Censored steps/day for 60 MVPA minutes (3 METs) among boys 6-11 years old (n=442). | | | | | | |
| --- | --- | --- | --- | --- | --- | --- |
| Steps/day | Specificity (False Positive) | Sensitivity (False Negative) | PPV | NPV | Accuracy |  |
| 6,500 | 1.00 (.00) | 0.94 (.06) | 1.00 | 0.47 | 0.94 |  |
| 7,000 | 1.00 (.00) | 0.91 (.09) | 1.00 | 0.37 | 0.91 |  |
| 7,500 | 1.00 (.00) | 0.88 (.12) | 1.00 | 0.30 | 0.89 |  |
| 8,000 | 1.00 (.00) | 0.83 (.17) | 1.00 | 0.24 | 0.84 |  |
| 8,500 | 1.00 (.00) | 0.78 (.22) | 1.00 | 0.19 | 0.79 |  |
| 9,000 | 1.00 (.00) | 0.72 (.28) | 1.00 | 0.16 | 0.73 |  |
| 9,500 | 1.00 (.00) | 0.66 (.34) | 1.00 | 0.13 | 0.68 |  |
| 10,000 | 1.00 (.00) | 0.58 (.42) | 1.00 | 0.11 | 0.60 |  |
| 10,500 | 1.00 (.00) | 0.49 (.51) | 1.00 | 0.09 | 0.52 |  |
| 11,000 | 1.00 (.00) | 0.44 (.56) | 1.00 | 0.09 | 0.47 |  |
| 11,500 | 1.00 (.00) | 0.37 (.63) | 1.00 | 0.08 | 0.40 |  |
| 12,000 | 1.00 (.00) | 0.32 (.68) | 1.00 | 0.07 | 0.35 |  |
| 12,500 | 1.00 (.00) | 0.27 (.73) | 1.00 | 0.07 | 0.31 |  |
| 13,000 | 1.00 (.00) | 0.24 (.76) | 1.00 | 0.06 | 0.28 |  |
| 13,500 | 1.00 (.00) | 0.20 (.80) | 1.00 | 0.06 | 0.24 |  |
| 14,000 | 1.00 (.00) | 0.16 (.84) | 1.00 | 0.06 | 0.20 |  |
| 14,500 | 1.00 (.00) | 0.13 (.87) | 1.00 | 0.06 | 0.17 |  |

| Additional file 1: Table S1b. Censored steps/day for 60 MVPA minutes (3 METs) among girls 6-11 years old (n=473). | | | | | | |
| --- | --- | --- | --- | --- | --- | --- |
| Steps/day | Specificity (False Positive) | Sensitivity (False Negative) | PPV | NPV | Accuracy |  |
| 6,500 | 1.00 (.00) | 0.85 (.15) | 1.00 | 0.12 | 0.85 |  |
| 7,000 | 1.00 (.00) | 0.79 (.21) | 1.00 | 0.09 | 0.79 |  |
| 7,500 | 1.00 (.00) | 0.74 (.26) | 1.00 | 0.07 | 0.75 |  |
| 8,000 | 1.00 (.00) | 0.68 (.32) | 1.00 | 0.06 | 0.69 |  |
| 8,500 | 1.00 (.00) | 0.62 (.38) | 1.00 | 0.05 | 0.63 |  |
| 9,000 | 1.00 (.00) | 0.54 (.46) | 1.00 | 0.04 | 0.55 |  |
| 9,500 | 1.00 (.00) | 0.48 (.52) | 1.00 | 0.04 | 0.49 |  |
| 10,000 | 1.00 (.00) | 0.42 (.58) | 1.00 | 0.03 | 0.43 |  |
| 10,500 | 1.00 (.00) | 0.34 (.66) | 1.00 | 0.03 | 0.35 |  |
| 11,000 | 1.00 (.00) | 0.26 (.74) | 1.00 | 0.03 | 0.27 |  |
| 11,500 | 1.00 (.00) | 0.19 (.81) | 1.00 | 0.02 | 0.21 |  |
| 12,000 | 1.00 (.00) | 0.17 (.83) | 1.00 | 0.02 | 0.19 |  |
| 12,500 | 1.00 (.00) | 0.14 (.86) | 1.00 | 0.02 | 0.16 |  |
| 13,000 | 1.00 (.00) | 0.11 (.89) | 1.00 | 0.02 | 0.13 |  |
| 13,500 | 1.00 (.00) | 0.08 (.92) | 1.00 | 0.02 | 0.10 |  |
| 14,000 | 1.00 (.00) | 0.05 (.95) | 1.00 | 0.02 | 0.07 |  |
| 14,500 | 1.00 (.00) | 0.03 (.97) | 1.00 | 0.02 | 0.05 |  |

PPV=positive predictive value. NPV = negative predictive value. Accuracy = true positives + true negatives / (true positives + true negatives + false positives + false negatives).

| Additional file 1: Table S2a. Censored steps/day for 60 MVPA minutes (4 METs) among boys 6-11 years old (n=442). | | | | | | |
| --- | --- | --- | --- | --- | --- | --- |
| Steps/day | Specificity (False Positive) | Sensitivity (False Negative) | PPV | NPV | Accuracy |  |
| 6,500 | 0.33 (.67) | 0.99 (.01) | 0.81 | 0.92 | 0.82 |  |
| 7,000 | 0.42 (.58) | 0.98 (.02) | 0.83 | 0.87 | 0.84 |  |
| 7,500 | 0.50 (.50) | 0.97 (.03) | 0.85 | 0.84 | 0.85 |  |
| 8,000 | 0.59 (.41) | 0.94 (.06) | 0.87 | 0.75 | 0.85 |  |
| 8,500 | 0.71 (.29) | 0.91 (.09) | 0.90 | 0.72 | 0.86 |  |
| 9,000 | 0.79 (.21) | 0.86 (.14) | 0.92 | 0.65 | 0.84 |  |
| 9,500 | 0.83 (.17) | 0.79 (.21) | 0.93 | 0.57 | 0.80 |  |
| 10,000 | 0.91 (.09) | 0.71 (.29) | 0.96 | 0.51 | 0.76 |  |
| 10,500 | 0.97 (.03) | 0.61 (.39) | 0.98 | 0.46 | 0.70 |  |
| 11,000 | 0.97 (.03) | 0.55 (.45) | 0.98 | 0.42 | 0.66 |  |
| 11,500 | 0.98 (.02) | 0.46 (.54) | 0.99 | 0.38 | 0.59 |  |
| 12,000 | 1.00 (.00) | 0.41 (.59) | 1.00 | 0.36 | 0.56 |  |
| 12,500 | 1.00 (.00) | 0.35 (.65) | 1.00 | 0.34 | 0.51 |  |
| 13,000 | 1.00 (.00) | 0.31 (.69) | 1.00 | 0.33 | 0.48 |  |
| 13,500 | 1.00 (.00) | 0.25 (.75) | 1.00 | 0.31 | 0.44 |  |
| 14,000 | 1.00 (.00) | 0.21 (.79) | 1.00 | 0.30 | 0.41 |  |
| 14,500 | 1.00 (.00) | 0.17 (.83) | 1.00 | 0.29 | 0.38 |  |

| Additional file 1: Table S2b. Censored steps/day for 60 MVPA minutes (4 METs) among girls 6-11 years old (n=473). | | | | | | |
| --- | --- | --- | --- | --- | --- | --- |
| Steps/day | Specificity (False Positive) | Sensitivity (False Negative) | PPV | NPV | Accuracy |  |
| 6,500 | 0.38 (.62) | 0.98 (.02) | 0.72 | 0.92 | 0.75 |  |
| 7,000 | 0.52 (.48) | 0.97 (.03) | 0.76 | 0.92 | 0.80 |  |
| 7,500 | 0.59 (.41) | 0.95 (.05) | 0.79 | 0.88 | 0.81 |  |
| 8,000 | 0.67 (.33) | 0.90 (.10) | 0.81 | 0.82 | 0.82 |  |
| 8,500 | 0.75 (.25) | 0.85 (.15) | 0.84 | 0.76 | 0.81 |  |
| 9,000 | 0.85 (.15) | 0.79 (.21) | 0.89 | 0.72 | 0.81 |  |
| 9,500 | 0.90 (.10) | 0.72 (.28) | 0.92 | 0.67 | 0.79 |  |
| 10,000 | 0.94 (.06) | 0.65 (.35) | 0.95 | 0.63 | 0.76 |  |
| 10,500 | 0.97 (.03) | 0.53 (.47) | 0.96 | 0.56 | 0.70 |  |
| 11,000 | 0.98 (.02) | 0.42 (.58) | 0.98 | 0.51 | 0.63 |  |
| 11,500 | 0.99 (.01) | 0.31 (.69) | 0.99 | 0.47 | 0.57 |  |
| 12,000 | 0.99 (.01) | 0.26 (.74) | 0.99 | 0.46 | 0.55 |  |
| 12,500 | 0.99 (.01) | 0.22 (.78) | 0.99 | 0.44 | 0.52 |  |
| 13,000 | 0.99 (.01) | 0.17 (.83) | 0.98 | 0.43 | 0.49 |  |
| 13,500 | 0.99 (.01) | 0.13 (.87) | 0.98 | 0.42 | 0.47 |  |
| 14,000 | 1.00 (.00) | 0.10 (.90) | 1.00 | 0.41 | 0.44 |  |
| 14,500 | 1.00 (.00) | 0.08 (.92) | 1.00 | 0.41 | 0.44 |  |

PPV=positive predictive value. NPV = negative predictive value. Accuracy = true positives + true negatives / (true positives + true negatives + false positives + false negatives).

| Additional file 1: Table S3a. Censored steps/day for 60 MVPA minutes (Evenson) among boys 6-11 years old (n=442). | | | | | | |
| --- | --- | --- | --- | --- | --- | --- |
| Steps/day | Specificity (False Positive) | Sensitivity (False Negative) | PPV | NPV | Accuracy |  |
| 6,500 | 0.16 (.84) | 1.00 (.00) | 0.50 | 1.00 | 0.54 |  |
| 7,000 | 0.22 (.78) | 1.00 (.00) | 0.52 | 1.00 | 0.58 |  |
| 7,500 | 0.26 (.74) | 1.00 (.00) | 0.53 | 0.98 | 0.60 |  |
| 8,000 | 0.34 (.66) | 0.99 (.01) | 0.56 | 0.97 | 0.64 |  |
| 8,500 | 0.42 (.58) | 0.98 (.02) | 0.59 | 0.95 | 0.68 |  |
| 9,000 | 0.51 (.49) | 0.95 (.05) | 0.62 | 0.93 | 0.71 |  |
| 9,500 | 0.59 (.41) | 0.91 (.09) | 0.65 | 0.89 | 0.74 |  |
| 10,000 | 0.72 (.28) | 0.89 (.11) | 0.73 | 0.88 | 0.80 |  |
| 10,500 | 0.81 (.19) | 0.80 (.20) | 0.78 | 0.83 | 0.81 |  |
| 11,000 | 0.85 (.15) | 0.74 (.26) | 0.81 | 0.79 | 0.80 |  |
| 11,500 | 0.92 (.08) | 0.67 (.33) | 0.87 | 0.77 | 0.80 |  |
| 12,000 | 0.95 (.05) | 0.61 (.39) | 0.91 | 0.74 | 0.80 |  |
| 12,500 | 0.97 (.03) | 0.54 (.46) | 0.94 | 0.71 | 0.77 |  |
| 13,000 | 0.98 (.02) | 0.48 (.52) | 0.95 | 0.69 | 0.75 |  |
| 13,500 | 0.98 (.02) | 0.40 (.60) | 0.95 | 0.66 | 0.71 |  |
| 14,000 | 1.00 (.00) | 0.34 (.66) | 0.99 | 0.64 | 0.69 |  |
| 14,500 | 1.00 (.00) | 0.27 (.73) | 0.98 | 0.62 | 0.66 |  |

| Additional file 1: Table S3b. Censored step/day for 60 MVPA minutes (Evenson) among girls 6-11 years old (n=473). | | | | | | |
| --- | --- | --- | --- | --- | --- | --- |
| Steps/day | Specificity (False Positive) | Sensitivity (False Negative) | PPV | NPV | Accuracy |  |
| 6,500 | 0.20 (.80) | 0.99 (.01) | 0.26 | 0.99 | 0.38 |  |
| 7,000 | 0.28 (.72) | 0.99 (.01) | 0.28 | 0.99 | 0.44 |  |
| 7,500 | 0.33 (.67) | 0.99 (.01) | 0.29 | 0.99 | 0.47 |  |
| 8,000 | 0.40 (.60) | 0.99 (.01) | 0.32 | 0.99 | 0.53 |  |
| 8,500 | 0.47 (.53) | 0.96 (.04) | 0.34 | 0.97 | 0.58 |  |
| 9,000 | 0.57 (.43) | 0.96 (.04) | 0.39 | 0.98 | 0.66 |  |
| 9,500 | 0.63 (.37) | 0.91 (.09) | 0.41 | 0.96 | 0.69 |  |
| 10,000 | 0.70 (.30) | 0.88 (.12) | 0.45 | 0.95 | 0.74 |  |
| 10,500 | 0.79 (.21) | 0.85 (.15) | 0.53 | 0.95 | 0.80 |  |
| 11,000 | 0.86 (.14) | 0.74 (.26) | 0.60 | 0.92 | 0.83 |  |
| 11,500 | 0.92 (.08) | 0.62 (.38) | 0.67 | 0.89 | 0.85 |  |
| 12,000 | 0.94 (.06) | 0.58 (.42) | 0.73 | 0.89 | 0.86 |  |
| 12,500 | 0.95 (.05) | 0.49 (.51) | 0.75 | 0.87 | 0.85 |  |
| 13,000 | 0.96 (.04) | 0.38 (.62) | 0.73 | 0.85 | 0.83 |  |
| 13,500 | 0.97 (.03) | 0.32 (.68) | 0.77 | 0.84 | 0.83 |  |
| 14,000 | 0.98 (.02) | 0.23 (.77) | 0.81 | 0.82 | 0.82 |  |
| 14,500 | 0.99 (.01) | 0.21 (.79) | 0.85 | 0.82 | 0.82 |  |

PPV=positive predictive value. NPV = negative predictive value. Accuracy = true positives + true negatives / (true positives + true negatives + false positives + false negatives).

| Additional file 1: Table S4a. Censored step/day for 60 MVPA minutes (3 METs) among boys 12-17 years old (n=646). | | | | | |
| --- | --- | --- | --- | --- | --- |
| Steps/day | Specificity (False Positive) | Sensitivity (False Negative) | PPV | NPV | Accuracy |
| 6,500 | 0.50 (.50) | 0.96 (.04) | 0.72 | 0.90 | 0.76 |
| 7,000 | 0.59 (.41) | 0.94 (.06) | 0.75 | 0.88 | 0.79 |
| 7,500 | 0.66 (.34) | 0.90 (.10) | 0.78 | 0.83 | 0.80 |
| 8,000 | 0.74 (.26) | 0.84 (.16) | 0.81 | 0.78 | 0.80 |
| 8,500 | 0.79 (.21) | 0.79 (.21) | 0.83 | 0.74 | 0.79 |
| 9,000 | 0.86 (.14) | 0.72 (.28) | 0.87 | 0.70 | 0.78 |
| 9,500 | 0.88 (.12) | 0.63 (.37) | 0.88 | 0.64 | 0.74 |
| 10,000 | 0.91 (.09) | 0.56 (.44) | 0.89 | 0.61 | 0.71 |
| 10,500 | 0.93 (.07) | 0.48 (.52) | 0.90 | 0.57 | 0.67 |
| 11,000 | 0.96 (.04) | 0.44 (.66) | 0.94 | 0.56 | 0.66 |
| 11,500 | 0.98 (.02) | 0.38 (.62) | 0.96 | 0.54 | 0.64 |
| 12,000 | 0.99 (.01) | 0.32 (.68) | 0.98 | 0.52 | 0.61 |
| 12,500 | 1.00 (.00) | 0.29 (.71) | 1.00 | 0.51 | 0.59 |
| 13,000 | 1.00 (.00) | 0.24 (.76) | 1.00 | 0.49 | 0.56 |
| 13,500 | 1.00 (.00) | 0.19 (.81) | 1.00 | 0.48 | 0.54 |
| 14,000 | 1.00 (.00) | 0.16 (.84) | 1.00 | 0.47 | 0.52 |
| 14,500 | 1.00 (.00) | 0.12 (.88) | 1.00 | 0.46 | 0.50 |

| Additional file 1: Table S4b. Censored steps/day for 60 MVPA minutes (3 METs) among girls 12-17 years old (n=656). | | | | | | | | | | | |
| --- | --- | --- | --- | --- | --- | --- | --- | --- | --- | --- | --- |
| Steps/day | | Specificity (False Positive) | Sensitivity (False Negative) | | | PPV | | NPV | | Accuracy | |
| 6,500 | | 0.52 (.48) | .98 (.02) | | | 0.44 | | 0.99 | | 0.65 | |
| 7,000 | | 0.69 (0.31) | 0.91 (.09) | | | 0.53 | | 0.95 | | 0.75 | |
| 7,500 | | 0.77 (0.23) | 0.84 (.16) | | | 0.58 | | 0.93 | | 0.79 | |
| 8,000 | | 0.83 (0.17) | 0.74 (.26) | | | 0.62 | | 0.89 | | 0.81 | |
| 8,500 | | 0.88 (0.12) | 0.65 (.35) | | | 0.67 | | 0.87 | | 0.82 | |
| 9,000 | | 0.92 (0.08) | 0.50 (.50) | | | 0.70 | | 0.83 | | 0.80 | |
| 9,500 | | 0.95 (0.05) | 0.43 (.57) | | | 0.76 | | 0.82 | | 0.81 | |
| 10,000 | | 0.97 (0.03) | 0.37 (.63) | | | 0.82 | | 0.80 | | 0.81 | |
| 10,500 | | 0.98 (0.02) | 0.32 (.68) | | | 0.86 | | 0.79 | | 0.80 | |
| 11,000 | | 1.00 (.00) | 0.26 (.74) | | | 1.00 | | 0.78 | | 0.80 | |
| 11,500 | | 1.00 (.00) | 0.22 (.78) | | | 1.00 | | 0.77 | | 0.79 | |
| 12,000 | | 1.00 (.00) | 0.18 (.82) | | | 1.00 | | 0.76 | | 0.78 | |
| 12,500 | | 1.00 (.00) | 0.14 (.86) | | | 1.00 | | 0.76 | | 0.76 | |
| 13,000 | | 1.00 (.00) | 0.13 (.87) | | | 1.00 | | 0.75 | | 0.76 | |
| 13,500 | | 1.00 (.00) | 0.10 (.90) | | | 1.00 | | 0.75 | | 0.75 | |
| 14,000 | | 1.00 (.00) | 0.08 (.92) | | | 1.00 | | 0.74 | | 0.75 | |
| 14,500 | | 1.00 (.00) | 0.06 (.94) | | | 1.00 | | 0.74 | | 0.74 | |
| PPV=positive predictive value. NPV = negative predictive value. Accuracy = true positives + true negatives / (true positives + true negatives + false positives + false negatives). | | | | | | | | | | | |
| Additional file 1: Table S5a. Censored steps/day for 60 MVPA minutes (4 METs) among boys 12-17 years old (n=646). | | | | | | | | | | |  |
| Steps/day | Specificity (False Positive) | | | Sensitivity (False Negative) | PPV | | NPV | | Accuracy | |  |
| 6,500 | 0.26 (.74) | | | 1.00 (.00) | 0.18 | | 1.00 | | 0.37 | |  |
| 7,000 | 0.32 (.68) | | | 1.00 (.00) | 0.20 | | 1.00 | | 0.42 | |  |
| 7,500 | 0.39 (.61) | | | 1.00 (.00) | 0.21 | | 1.00 | | 0.47 | |  |
| 8,000 | 0.47 (.53) | | | 1.00 (.00) | 0.24 | | 1.00 | | 0.54 | |  |
| 8,500 | 0.53 (.47) | | | 1.00 (.00) | 0.26 | | 1.00 | | 0.60 | |  |
| 9,000 | 0.61 (.39) | | | 0.99 (.01) | 0.30 | | 1.00 | | 0.67 | |  |
| 9,500 | 0.68 (.32) | | | 0.95 (.05) | 0.33 | | 0.99 | | 0.72 | |  |
| 10,000 | 0.74 (.26) | | | 0.93 (.07) | 0.37 | | 0.98 | | 0.76 | |  |
| 10,500 | 0.80 (.20) | | | 0.90 (.10) | 0.43 | | 0.98 | | 0.81 | |  |
| 11,000 | 0.84 (16) | | | 0.88 (.12) | 0.49 | | 0.98 | | 0.85 | |  |
| 11,500 | 0.89 (.11) | | | 0.86 (.14) | 0.56 | | 0.97 | | 0.88 | |  |
| 12,000 | 0.92 (.08) | | | 0.78 (.22) | 0.61 | | 0.96 | | 0.90 | |  |
| 12,500 | 0.94 (.06) | | | 0.72 (.28) | 0.65 | | 0.95 | | 0.90 | |  |
| 13,000 | 0.96 (.04) | | | 0.64 (.36) | 0.70 | | 0.94 | | 0.91 | |  |
| 13,500 | 0.97 (.03) | | | 0.51 (.49) | 0.71 | | 0.92 | | 0.90 | |  |
| 14,000 | 0.98 (.02) | | | 0.47 (.53) | 0.78 | | 0.92 | | 0.91 | |  |
| 14,500 | 0.98 (.02) | | | 0.42 (.58) | 0.78 | | 0.91 | | 0.90 | |  |

| Additional file 1: Table S5b. Censored step/day for 60 MVPA minutes (4 METs) among girls 12-17 years old (n=656). | | | | | |
| --- | --- | --- | --- | --- | --- |
| Steps/day | Specificity (False Positive) | Sensitivity (False Negative) | PPV | NPV | Accuracy |
| 6,500 | 0.45 (.55) | 0.95 (.05) | 0.05 | 1.00 | 0.46 |
| 7,000 | 0.52 (.48) | 0.91 (.09) | 0.05 | 0.99 | 0.53 |
| 7,500 | 0.60 (.40) | 0.86 (.14) | 0.06 | 0.99 | 0.61 |
| 8,000 | 0.67 (.33) | 0.86 (.14) | 0.07 | 0.99 | 0.67 |
| 8,500 | 0.73 (.27) | 0.86 (.14) | 0.09 | 0.99 | 0.74 |
| 9,000 | 0.82 (.18) | 0.81 (.19) | 0.12 | 0.99 | 0.82 |
| 9,500 | 0.86 (.14) | 0.81 (.19) | 0.15 | 0.99 | 0.86 |
| 10,000 | 0.89 (.11) | 0.76 (.24) | 0.17 | 0.99 | 0.88 |
| 10,500 | 0.91 (.09) | 0.71 (.29) | 0.19 | 0.99 | 0.90 |
| 11,000 | 0.94 (.06) | 0.62 (.38) | 0.23 | 0.99 | 0.93 |
| 11,500 | 0.95 (.05) | 0.57 (.43) | 0.25 | 0.99 | 0.94 |
| 12,000 | 0.96 (.04) | 0.48 (.52) | 0.27 | 0.98 | 0.95 |
| 12,500 | 0.97 (.03) | 0.43 (.57) | 0.29 | 0.98 | 0.95 |
| 13,000 | 0.97 (.03) | 0.33 (.67) | 0.25 | 0.98 | 0.95 |
| 13,500 | 0.98 (.02) | 0.24 (.76) | 0.23 | 0.98 | 0.95 |
| 14,000 | 0.98 (.02) | 0.24 (.76) | 0.31 | 0.98 | 0.96 |
| 14,500 | 0.99 (.01) | 0.19 (.81) | 0.34 | 0.98 | 0.97 |

PPV=positive predictive value. NPV = negative predictive value. Accuracy = true positives + true negatives / (true positives + true negatives + false positives + false negatives).

| Additional file 1: Table S6a. Censored steps/day for 60 MVPA minutes (Evenson) among boys 12-17 years old (n=646). | | | | | | |
| --- | --- | --- | --- | --- | --- | --- |
| Steps/day | Specificity (False Positive) | Sensitivity (False Negative) | PPV | NPV | Accuracy |  |
| 6,500 | 0.30 (.70) | 1.00 (.00) | 0.29 | 1.00 | 0.45 |  |
| 7,000 | 0.36 (.64) | 1.00 (.00) | 0.31 | 1.00 | 0.50 |  |
| 7,500 | 0.43 (.57) | 0.99 (.01) | 0.33 | 1.00 | 0.56 |  |
| 8,000 | 0.52 (.48) | 0.99 (.01) | 0.37 | 0.99 | 0.62 |  |
| 8,500 | 0.59 (.41) | 0.98 (.02) | 0.41 | 0.99 | 0.68 |  |
| 9,000 | 0.68 (.32) | 0.95 (.05) | 0.45 | 0.98 | 0.74 |  |
| 9,500 | 0.74 (.26) | 0.91 (.09) | 0.50 | 0.97 | 0.78 |  |
| 10,000 | 0.79 (.21) | 0.85 (.15) | 0.53 | 0.95 | 0.80 |  |
| 10,500 | 0.85 (.15) | 0.79 (.21) | 0.59 | 0.93 | 0.83 |  |
| 11,000 | 0.89 (.11) | 0.75 (.25) | 0.65 | 0.93 | 0.86 |  |
| 11,500 | 0.93 (.07) | 0.72 (.28) | 0.75 | 0.92 | 0.88 |  |
| 12,000 | 0.97 (.03) | 0.66 (.34) | 0.85 | 0.91 | 0.90 |  |
| 12,500 | 0.98 (.02) | 0.60 (.40) | 0.88 | 0.90 | 0.89 |  |
| 13,000 | 0.98 (.02) | 0.51 (.49) | 0.90 | 0.88 | 0.88 |  |
| 13,500 | 0.99 (.01) | 0.42 (.58) | 0.93 | 0.86 | 0.87 |  |
| 14,000 | 1.00 (.00) | 0.36 (.64) | 0.96 | 0.85 | 0.86 |  |
| 14,500 | 1.00 (.00) | 0.34 (.66) | 0.98 | 0.84 | 0.85 |  |

| Additional file 1: Table S6b. Censored steps/day for 60 MVPA minutes (Evenson) among girls 12-17 years old (n=656). | | | | | | |
| --- | --- | --- | --- | --- | --- | --- |
| Steps/day | Specificity (False Positive) | Sensitivity (False Negative) | PPV | NPV | Accuracy |  |
| 6,500 | 0.47 (.53) | 0.97 (.03) | 0.12 | 1.00 | 0.50 |  |
| 7,000 | 0.53 (.47) | 0.95 (.05) | 0.13 | 0.99 | 0.56 |  |
| 7,500 | 0.61 (.39) | 0.92 (.08) | 0.15 | 0.99 | 0.63 |  |
| 8,000 | 0.69 (.31) | 0.92 (.08) | 0.17 | 0.99 | 0.70 |  |
| 8,500 | 0.76 (.24) | 0.92 (.08) | 0.21 | 0.99 | 0.77 |  |
| 9,000 | 0.84 (.16) | 0.90 (.10) | 0.28 | 0.99 | 0.84 |  |
| 9,500 | 0.88 (.12) | 0.87 (.13) | 0.34 | 0.99 | 0.88 |  |
| 10,000 | 0.91 (.09) | 0.85 (.15) | 0.40 | 0.99 | 0.91 |  |
| 10,500 | 0.93 (.07) | 0.79 (.21) | 0.45 | 0.98 | 0.92 |  |
| 11,000 | 0.96 (.04) | 0.74 (.26) | 0.59 | 0.98 | 0.95 |  |
| 11,500 | 0.97 (.03) | 0.69 (.31) | 0.64 | 0.98 | 0.95 |  |
| 12,000 | 0.98 (.02) | 0.59 (.41) | 0.68 | 0.97 | 0.95 |  |
| 12,500 | 0.99 (.01) | 0.51 (.49) | 0.71 | 0.97 | 0.95 |  |
| 13,000 | 0.99 (.01) | 0.49 (.51) | 0.75 | 0.96 | 0.96 |  |
| 13,500 | 0.99 (.01) | 0.38 (.62) | 0.77 | 0.96 | 0.95 |  |
| 14,000 | 0.99 (.01) | 0.28 (.72) | 0.76 | 0.95 | 0.95 |  |
| 14,500 | 1.00 (.00) | 0.23 (.77) | 0.77 | 0.95 | 0.94 |  |

PPV=positive predictive value. NPV = negative predictive value. Accuracy = true positives + true negatives / (true positives + true negatives + false positives + false negatives).

| Additional file 1: Table S7a. Uncensored steps/day for 60 MVPA minutes (3 METs) among boys 6-11 years old (n=442). | | | | | | |
| --- | --- | --- | --- | --- | --- | --- |
| Steps/day | Specificity (False Positive) | Sensitivity (False Negative) | PPV | NPV | Accuracy |  |
| 6,500 | 0.64 (36) | 0.99 (.01) | 0.98 | 0.83 | 0.98 |  |
| 7,000 | 0.73 (.27) | 0.98 (.02) | 0.99 | 0.67 | 0.97 |  |
| 7,500 | 0.91 (.09) | 0.97 (.03) | 1.00 | 0.65 | 0.97 |  |
| 8,000 | 0.91 (.09) | 0.96 (.04) | 1.00 | 0.55 | 0.96 |  |
| 8,500 | 0.91 (.09) | 0.94 (.06) | 0.99 | 0.46 | 0.94 |  |
| 9,000 | 1.00 (.00) | 0.93 (.07) | 1.00 | 0.42 | 0.93 |  |
| 9,500 | 1.00 (.00) | 0.90 (.10) | 1.00 | 0.35 | 0.91 |  |
| 10,000 | 1.00 (.00) | 0.86 (.14) | 1.00 | 0.27 | 0.87 |  |
| 10,500 | 1.00 (.00) | 0.83 (.17) | 1.00 | 0.23 | 0.83 |  |
| 11,000 | 1.00 (.00) | 0.78 (.22) | 1.00 | 0.20 | 0.79 |  |
| 11,500 | 1.00 (.00) | 0.72 (.28) | 1.00 | 0.16 | 0.74 |  |
| 12,000 | 1.00 (.00) | 0.67 (.33) | 1.00 | 0.14 | 0.68 |  |
| 12,500 | 1.00 (.00) | 0.60 (.40) | 1.00 | 0.12 | 0.62 |  |
| 13,000 | 1.00 (.00) | 0.52 (.48) | 1.00 | 0.10 | 0.54 |  |
| 13,500 | 1.00 (.00) | 0.44 (.56) | 1.00 | 0.09 | 0.47 |  |
| 14,000 | 1.00 (.00) | 0.39 (.61) | 1.00 | 0.08 | 0.42 |  |
| 14,500 | 1.00 (.00) | 0.34 (.66) | 1.00 | 0.07 | 0.37 |  |

| Additional file 1: Table S7b. Uncensored steps/day for 60 MVPA minutes (3 METs) among girls 6-11 years old (n=473). | | | | | | |
| --- | --- | --- | --- | --- | --- | --- |
| Steps/day | Specificity (False Positive) | Sensitivity (False Negative) | PPV | NPV | Accuracy |  |
| 6,500 | 0.57 (.43) | 1.00 (.00) | 0.99 | 0.73 | 0.99 |  |
| 7,000 | 0.57 (.43) | 0.99 (.01) | 0.99 | 0.47 | 0.98 |  |
| 7,500 | 0.57 (.43) | 0.97 (.03) | 0.99 | 0.25 | 0.96 |  |
| 8,000 | 0.71 (.29) | 0.94 (.06) | 0.99 | 0.20 | 0.94 |  |
| 8,500 | 1.00 (.00) | 0.91 (.09) | 1.00 | 0.18 | 0.91 |  |
| 9,000 | 1.00 (.00) | 0.86 (.14) | 1.00 | 0.12 | 0.86 |  |
| 9,500 | 1.00 (.00) | 0.82 (.18) | 1.00 | 0.10 | 0.82 |  |
| 10,000 | 1.00 (.00) | 0.76 (.24) | 1.00 | 0.08 | 0.77 |  |
| 10,500 | 1.00 (.00) | 0.71 (.29) | 1.00 | 0.06 | 0.71 |  |
| 11,000 | 1.00 (.00) | 0.65 (.35) | 1.00 | 0.05 | 0.66 |  |
| 11,500 | 1.00 (.00) | 0.59 (.41) | 1.00 | 0.05 | 0.60 |  |
| 12,000 | 1.00 (.00) | 0.52 (.48) | 1.00 | 0.04 | 0.53 |  |
| 12,500 | 1.00 (.00) | 0.45 (.55) | 1.00 | 0.04 | 0.46 |  |
| 13,000 | 1.00 (.00) | 0.39 (.61) | 1.00 | 0.03 | 0.41 |  |
| 13,500 | 1.00 (.00) | 0.32 (.68) | 1.00 | 0.03 | 0.34 |  |
| 14,000 | 1.00 (.00) | 0.25 (.75) | 1.00 | 0.03 | 0.27 |  |
| 14,500 | 1.00 (.00) | 0.21 (.79) | 1.00 | 0.02 | 0.23 |  |

PPV=positive predictive value. NPV = negative predictive value. Accuracy = true positives + true negatives / (true positives + true negatives + false positives + false negatives).

| Additional file 1: Table S8a. Uncensored steps/day for 60 MVPA minutes (4 METs) among boys 6-11 years old (n=442). | | | | | | |
| --- | --- | --- | --- | --- | --- | --- |
| Steps/day | Specificity (False Positive) | Sensitivity (False Negative) | PPV | NPV | Accuracy |  |
| 6,500 | 0.10 (.90) | 1.00 (.00) | 0.77 | 1.00 | 0.77 |  |
| 7,000 | 0.14 (.86) | 1.00 (.00) | 0.78 | 0.94 | 0.78 |  |
| 7,500 | 0.19 (.81) | 1.00 (.00) | 0.79 | 0.96 | 0.79 |  |
| 8,000 | 0.25 (.75) | 1.00 (.00) | 0.80 | 0.97 | 0.81 |  |
| 8,500 | 0.30 (.70) | 0.99 (.01) | 0.81 | 0.92 | 0.82 |  |
| 9,000 | 0.34 (.66) | 0.98 (.02) | 0.81 | 0.85 | 0.82 |  |
| 9,500 | 0.41 (.59) | 0.97 (.03) | 0.83 | 0.83 | 0.83 |  |
| 10,000 | 0.49 (.51) | 0.94 (.06) | 0.84 | 0.73 | 0.82 |  |
| 10,500 | 0.56 (.44) | 0.92 (.08) | 0.86 | 0.69 | 0.83 |  |
| 11,000 | 0.67 (.33) | 0.90 (.10) | 0.89 | 0.69 | 0.84 |  |
| 11,500 | 0.75 (.25) | 0.85 (.15) | 0.91 | 0.62 | 0.82 |  |
| 12,000 | 0.80 (.20) | 0.79 (.21) | 0.92 | 0.56 | 0.79 |  |
| 12,500 | 0.87 (.13) | 0.72 (.28) | 0.94 | 0.52 | 0.76 |  |
| 13,000 | 0.91 (.09) | 0.63 (.37) | 0.96 | 0.46 | 0.70 |  |
| 13,500 | 0.95 (.05) | 0.55 (.45) | 0.97 | 0.42 | 0.65 |  |
| 14,000 | 0.98 (.02) | 0.50 (.50) | 0.99 | 0.40 | 0.62 |  |
| 14,500 | 0.98 (.02) | 0.43 (.57) | 0.98 | 0.37 | 0.57 |  |

| Additional file 1: Table S8b. Uncensored steps/day for 60 MVPA minutes (4 METs) among girls 6-11 years old (n=473). | | | | | | |
| --- | --- | --- | --- | --- | --- | --- |
| Steps/day | Specificity (False Positive) | Sensitivity (False Negative) | PPV | NPV | Accuracy |  |
| 6,500 | 0.03 (.97) | 1.00 (.00) | 0.62 | 1.00 | 0.63 |  |
| 7,000 | 0.05 (.95) | 1.00 (.00) | 0.63 | 0.89 | 0.63 |  |
| 7,500 | 0.09 (.91) | 0.99 (.01) | 0.63 | 0.84 | 0.64 |  |
| 8,000 | 0.15 (.85) | 0.99 (.01) | 0.65 | 0.87 | 0.66 |  |
| 8,500 | 0.24 (.76) | 0.99 (.01) | 0.67 | 0.91 | 0.70 |  |
| 9,000 | 0.34 (.66) | 0.97 (.03) | 0.70 | 0.88 | 0.73 |  |
| 9,500 | 0.43 (.57) | 0.96 (.04) | 0.73 | 0.88 | 0.76 |  |
| 10,000 | 0.54 (.66) | 0.95 (.05) | 0.77 | 0.87 | 0.79 |  |
| 10,500 | 0.62 (.38) | 0.90 (.10) | 0.79 | 0.80 | 0.79 |  |
| 11,000 | 0.69 (.31) | 0.87 (.13) | 0.82 | 0.77 | 0.80 |  |
| 11,500 | 0.76 (.24) | 0.81 (.19) | 0.84 | 0.71 | 0.79 |  |
| 12,000 | 0.83 (.17) | 0.73 (.27) | 0.87 | 0.66 | 0.77 |  |
| 12,500 | 0.88 (.12) | 0.66 (.34) | 0.90 | 0.62 | 0.75 |  |
| 13,000 | 0.93 (.07) | 0.60 (.40) | 0.93 | 0.59 | 0.72 |  |
| 13,500 | 0.96 (.04) | 0.50 (.50) | 0.95 | 0.55 | 0.68 |  |
| 14,000 | 0.97 (.03) | 0.40 (.60) | 0.96 | 0.50 | 0.62 |  |
| 14,500 | 0.99 (.01) | 0.34 (.66) | 0.98 | 0.48 | 0.59 |  |

PPV=positive predictive value. NPV = negative predictive value. Accuracy = true positives + true negatives / (true positives + true negatives + false positives + false negatives).

| Additional file 1: Table S9a. Uncensored steps/day for 60 MVPA minutes (Evenson) among boys 6-11 years old (n=442). | | | | | | |
| --- | --- | --- | --- | --- | --- | --- |
| Steps/day | Specificity (False Positive) | Sensitivity (False Negative) | PPV | NPV | Accuracy |  |
| 6,500 | 0.04 (.96) | 1.00 (.00) | 0.47 | 1.00 | 0.48 |  |
| 7,000 | 0.07 (.93) | 1.00 (.00) | 0.48 | 1.00 | 0.50 |  |
| 7,500 | 0.09 (.91) | 1.00 (.00) | 0.48 | 1.00 | 0.51 |  |
| 8,000 | 0.11 (.89) | 1.00 (.00) | 0.49 | 1.00 | 0.52 |  |
| 8,500 | 0.14 (.86) | 1.00 (.00) | 0.50 | 1.00 | 0.54 |  |
| 9,000 | 0.18 (.82) | 1.00 (.00) | 0.51 | 1.00 | 0.55 |  |
| 9,500 | 0.22 (.78) | 1.00 (.00) | 0.52 | 1.00 | 0.58 |  |
| 10,000 | 0.28 (.72) | 0.98 (.02) | 0.54 | 0.93 | 0.60 |  |
| 10,500 | 0.33 (.67) | 0.97 (.03) | 0.55 | 0.92 | 0.62 |  |
| 11,000 | 0.41 (.59) | 0.97 (.03) | 0.58 | 0.93 | 0.66 |  |
| 11,500 | 0.49 (.51) | 0.93 (.07) | 0.61 | 0.89 | 0.69 |  |
| 12,000 | 0.57 (.43) | 0.91 (.09) | 0.64 | 0.88 | 0.72 |  |
| 12,500 | 0.66 (.34) | 0.87 (.13) | 0.68 | 0.86 | 0.75 |  |
| 13,000 | 0.74 (.26) | 0.79 (.21) | 0.72 | 0.81 | 0.76 |  |
| 13,500 | 0.81 (.19) | 0.72 (.28) | 0.77 | 0.77 | 0.77 |  |
| 14,000 | 0.86 (.14) | 0.67 (.33) | 0.81 | 0.76 | 0.78 |  |
| 14,500 | 0.91 (.09) | 0.61 (.39) | 0.85 | 0.73 | 0.77 |  |

| Additional file 1: Table S9b. Uncensored steps/day for 60 MVPA minutes (Evenson) among girls 6-11 years old (n=473). | | | | | | |
| --- | --- | --- | --- | --- | --- | --- |
| Steps/day | Specificity (False Positive) | Sensitivity (False Negative) | PPV | NPV | Accuracy |  |
| 6,500 | 0.02 (.98) | 1.00 (.00) | 0.22 | 1.00 | 0.23 |  |
| 7,000 | 0.03 (.97) | 1.00 (.00) | 0.22 | 1.00 | 0.24 |  |
| 7,500 | 0.05 (.95) | 0.99 (.01) | 0.23 | 0.94 | 0.26 |  |
| 8,000 | 0.08 (.92) | 0.99 (.01) | 0.23 | 0.96 | 0.28 |  |
| 8,500 | 0.13 (.87) | 0.99 (.01) | 0.24 | 0.98 | 0.32 |  |
| 9,000 | 0.19 (.81) | 0.99 (.01) | 0.26 | 0.98 | 0.36 |  |
| 9,500 | 0.24 (.76) | 0.99 (.01) | 0.27 | 0.99 | 0.41 |  |
| 10,000 | 0.30 (.70) | 0.99 (.01) | 0.29 | 0.99 | 0.45 |  |
| 10,500 | 0.37 (.63) | 0.97 (.03) | 0.30 | 0.98 | 0.50 |  |
| 11,000 | 0.43 (.57) | 0.96 (.04) | 0.32 | 0.97 | 0.55 |  |
| 11,500 | 0.50 (.50) | 0.93 (.07) | 0.35 | 0.96 | 0.60 |  |
| 12,000 | 0.58 (.42) | 0.89 (.11) | 0.37 | 0.95 | 0.65 |  |
| 12,500 | 0.65 (.35) | 0.86 (.14) | 0.41 | 0.94 | 0.70 |  |
| 13,000 | 0.71 (.29) | 0.82 (.18) | 0.45 | 0.94 | 0.74 |  |
| 13,500 | 0.78 (.22) | 0.73 (.27) | 0.48 | 0.91 | 0.77 |  |
| 14,000 | 0.85 (.15) | 0.66 (.34) | 0.55 | 0.90 | 0.81 |  |
| 14,500 | 0.89 (.11) | 0.62 (.38) | 0.61 | 0.89 | 0.83 |  |

PPV=positive predictive value. NPV = negative predictive value. Accuracy = true positives + true negatives / (true positives + true negatives + false positives + false negatives).

| Additional file 1: Table S10a. Uncensored steps/day for 60 MVPA minutes (3 METs) among boys 12-17 years old (n=646). | | | | | |
| --- | --- | --- | --- | --- | --- |
| Steps/day | Specificity (False Positive) | Sensitivity (False Negative) | PPV | NPV | Accuracy |
| 6,500 | 0.14 (.86) | 1.00 (.00) | 0.61 | 0.98 | 0.63 |
| 7,000 | 0.20 (.80) | 1.00 (.00) | 0.63 | 0.98 | 0.66 |
| 7,500 | 0.26 (.74) | 0.99 (.01) | 0.64 | 0.95 | 0.68 |
| 8,000 | 0.34 (.66) | 0.98 (.02) | 0.67 | 0.93 | 0.71 |
| 8,500 | 0.41 (.59) | 0.97 (.03) | 0.69 | 0.92 | 0.73 |
| 9,000 | 0.47 (.53) | 0.97 (.03) | 0.71 | 0.91 | 0.75 |
| 9,500 | 0.54 (.46) | 0.92 (.08) | 0.73 | 0.83 | 0.76 |
| 10,000 | 0.61 (.39) | 0.87 (.13) | 0.75 | 0.77 | 0.76 |
| 10,500 | 0.68 (.32) | 0.82 (.18) | 0.77 | 0.73 | 0.76 |
| 11,000 | 0.72 (.28) | 0.76 (.24) | 0.78 | 0.69 | 0.74 |
| 11,500 | 0.80 (.20) | 0.69 (.31) | 0.82 | 0.66 | 0.74 |
| 12,000 | 0.84 (.16) | 0.62 (.38) | 0.84 | 0.62 | 0.72 |
| 12,500 | 0.87 (.13) | 0.57 (.43) | 0.85 | 0.60 | 0.70 |
| 13,000 | 0.90 (.10) | 0.53 (.47) | 0.87 | 0.59 | 0.69 |
| 13,500 | 0.92 (.08) | 0.47 (.53) | 0.89 | 0.57 | 0.67 |
| 14,000 | 0.96 (.04) | 0.42 (.58) | 0.93 | 0.55 | 0.65 |
| 14,500 | 0.98 (.02) | 0.35 (.65) | 0.95 | 0.53 | 0.62 |

| Additional file 1: Table S10b. Uncensored steps/day for 60 MVPA minutes (3 METs) among girls 12-17 years old (n=656). | | | | | | | | | | | |
| --- | --- | --- | --- | --- | --- | --- | --- | --- | --- | --- | --- |
| Steps/day | | Specificity (False Positive) | Sensitivity (False Negative) | | | PPV | | NPV | | Accuracy | |
| 6,500 | | 0.17 (.83) | 1.00 (.00) | | | 0.31 | | 0.99 | | 0.40 | |
| 7,000 | | 0.23 (.77) | 0.99 (.01) | | | 0.33 | | 0.98 | | 0.44 | |
| 7,500 | | 0.30 (.70) | 0.99 (.01) | | | 0.35 | | 0.98 | | 0.48 | |
| 8,000 | | 0.38 (.62) | 0.99 (.01) | | | 0.38 | | 0.99 | | 0.55 | |
| 8,500 | | 0.47 (.53) | 0.96 (.04) | | | 0.41 | | 0.97 | | 0.61 | |
| 9,000 | | 0.56 (.44) | 0.92 (.08) | | | 0.44 | | 0.95 | | 0.66 | |
| 9,500 | | 0.63 (.37) | 0.88 (.12) | | | 0.48 | | 0.93 | | 0.70 | |
| 10,000 | | 0.71 (.29) | 0.82 (.18) | | | 0.52 | | 0.91 | | 0.74 | |
| 10,500 | | 0.76 (.24) | 0.76 (.24) | | | 0.55 | | 0.89 | | 0.76 | |
| 11,000 | | 0.83 (.17) | 0.64 (.36) | | | 0.58 | | 0.86 | | 0.78 | |
| 11,500 | | 0.88 (.12) | 0.53 (.47) | | | 0.62 | | 0.83 | | 0.78 | |
| 12,000 | | 0.91 (.09) | 0.48 (.52) | | | 0.67 | | 0.82 | | 0.79 | |
| 12,500 | | 0.94 (.06) | 0.39 (.61) | | | 0.72 | | 0.80 | | 0.79 | |
| 13,000 | | 0.96 (.04) | 0.34 (.66) | | | 0.76 | | 0.79 | | 0.79 | |
| 13,500 | | 0.98 (.02) | 0.28 (.72) | | | 0.84 | | 0.78 | | 0.79 | |
| 14,000 | | 0.98 (.02) | 0.24 (.76) | | | 0.85 | | 0.77 | | 0.78 | |
| 14,500 | | 1.00 (.01) | 0.20 (.80) | | | 0.97 | | 0.77 | | 0.78 | |
| PPV=positive predictive value. NPV = negative predictive value. Accuracy = true positives + true negatives / (true positives + true negatives + false positives + false negatives). | | | | | | | | | | | |
| Additional file 1: Table S11a. Uncensored steps/day for 60 MVPA minutes (4 METs) among boys 12-17 years old (n=646). | | | | | | | | | | |  |
| Steps/day | Specificity (False Positive) | | | Sensitivity (False Negative) | PPV | | NPV | | Accuracy | |  |
| 6,500 | 0.07 (.93) | | | 1.00 (.00) | 0.15 | | 1.00 | | 0.20 | |  |
| 7,000 | 0.09 (.91) | | | 1.00 (.00) | 0.16 | | 1.00 | | 0.22 | |  |
| 7,500 | 0.13 (.87) | | | 1.00 (.00) | 0.16 | | 1.00 | | 0.25 | |  |
| 8,000 | 0.17 (.83) | | | 1.00 (.00) | 0.17 | | 1.00 | | 0.29 | |  |
| 8,500 | 0.21 (.79) | | | 1.00 (.00) | 0.17 | | 1.00 | | 0.32 | |  |
| 9,000 | 0.25 (.75) | | | 1.00 (.00) | 0.18 | | 1.00 | | 0.35 | |  |
| 9,500 | 0.31 (.69) | | | 1.00 (.00) | 0.20 | | 1.00 | | 0.41 | |  |
| 10,000 | 0.39 (.61) | | | 0.99 (.01) | 0.21 | | 1.00 | | 0.47 | |  |
| 10,500 | 0.45 (.55) | | | 0.99 (.01) | 0.23 | | 1.00 | | 0.53 | |  |
| 11,000 | 0.51 (.49) | | | 0.98 (.02) | 0.25 | | 0.99 | | 0.58 | |  |
| 11,500 | 0.59 (.41) | | | 0.94 (.06) | 0.27 | | 0.98 | | 0.64 | |  |
| 12,000 | 0.66 (.34) | | | 0.94 (.06) | 0.32 | | 0.98 | | 0.70 | |  |
| 12,500 | 0.71 (.29) | | | 0.91 (.09) | 0.34 | | 0.98 | | 0.73 | |  |
| 13,000 | 0.74 (.26) | | | 0.91 (.09) | 0.37 | | 0.98 | | 0.77 | |  |
| 13,500 | 0.79 (.21) | | | 0.86 (.14) | 0.41 | | 0.97 | | 0.80 | |  |
| 14,000 | 0.85 (.15) | | | 0.83 (.17) | 0.47 | | 0.97 | | 0.84 | |  |
| 14,500 | 0.89 (.11) | | | 0.78 (.22) | 0.54 | | 0.96 | | 0.87 | |  |

| Additional file 1: Table S11b. Uncensored steps/day for 60 MVPA minutes (4 METs) among girls 12-17 years old (n=656). | | | | | |
| --- | --- | --- | --- | --- | --- |
| Steps/day | Specificity (False Positive) | Sensitivity (False Negative) | PPV | NPV | Accuracy |
| 6,500 | 0.12 (.88) | 0.95 (.05) | 0.03 | 0.99 | 0.15 |
| 7,000 | 0.17 (.83) | 0.95 (.05) | 0.03 | 0.99 | 0.19 |
| 7,500 | 0.21 (.79) | 0.95 (.05) | 0.03 | 0.99 | 0.24 |
| 8,000 | 0.28 (.72) | 0.95 (.05) | 0.04 | 0.99 | 0.30 |
| 8,500 | 0.35 (.65) | 0.90 (.10) | 0.04 | 0.99 | 0.36 |
| 9,000 | 0.43 (.57) | 0.90 (.10) | 0.04 | 0.99 | 0.44 |
| 9,500 | 0.49 (.51) | 0.86 (.14) | 0.05 | 0.99 | 0.50 |
| 10,000 | 0.56 (.44) | 0.86 (.14) | 0.05 | 0.99 | 0.57 |
| 10,500 | 0.62 (.38) | 0.86 (.14) | 0.06 | 0.99 | 0.62 |
| 11,000 | 0.70 (.30) | 0.86 (.14) | 0.08 | 0.99 | 0.70 |
| 11,500 | 0.77 (.23) | 0.81 (.19) | 0.09 | 0.99 | 0.77 |
| 12,000 | 0.81 (.19) | 0.76 (.24) | 0.11 | 0.99 | 0.81 |
| 12,500 | 0.86 (.14) | 0.71 (.29) | 0.13 | 0.99 | 0.85 |
| 13,000 | 0.89 (.11) | 0.67 (.33) | 0.15 | 0.99 | 0.88 |
| 13,500 | 0.92 (.08) | 0.62 (.38) | 0.18 | 0.99 | 0.91 |
| 14,000 | 0.93 (.07) | 0.62 (.38) | 0.22 | 0.99 | 0.92 |
| 14,500 | 0.95 (.05) | 0.52 (.48) | 0.24 | 0.99 | 0.94 |

PPV=positive predictive value. NPV = negative predictive value. Accuracy = true positives + true negatives / (true positives + true negatives + false positives + false negatives).

| Additional file 1: Table S12a. Uncensored steps/day for 60 MVPA minutes (Evenson) among boys 12-17 years old (n=646). | | | | | | |
| --- | --- | --- | --- | --- | --- | --- |
| Steps/day | Specificity (False Positive) | Sensitivity (False Negative) | PPV | NPV | Accuracy |  |
| 6,500 | 0.08 (.92) | 1.00 (.00) | 0.23 | 1.00 | 0.28 |  |
| 7,000 | 0.11 (.89) | 1.00 (.00) | 0.24 | 1.00 | 0.30 |  |
| 7,500 | 0.15 (.85) | 1.00 (.00) | 0.25 | 1.00 | 0.34 |  |
| 8,000 | 0.19 (.81) | 1.00 (.00) | 0.26 | 1.00 | 0.37 |  |
| 8,500 | 0.24 (.76) | 1.00 (.00) | 0.27 | 1.00 | 0.41 |  |
| 9,000 | 0.28 (.72) | 1.00 (.00) | 0.28 | 1.00 | 0.44 |  |
| 9,500 | 0.35 (.65) | 0.99 (.01) | 0.30 | 0.99 | 0.49 |  |
| 10,000 | 0.43 (.57) | 0.98 (.02) | 0.33 | 0.98 | 0.55 |  |
| 10,500 | 0.50 (.50) | 0.96 (.04) | 0.35 | 0.98 | 0.60 |  |
| 11,000 | 0.56 (.44) | 0.94 (.06) | 0.38 | 0.97 | 0.64 |  |
| 11,500 | 0.64 (.36) | 0.91 (.09) | 0.42 | 0.96 | 0.70 |  |
| 12,000 | 0.71 (.29) | 0.87 (.13) | 0.46 | 0.95 | 0.75 |  |
| 12,500 | 0.76 (.24) | 0.84 (.16) | 0.49 | 0.94 | 0.78 |  |
| 13,000 | 0.79 (.21) | 0.81 (.19) | 0.52 | 0.94 | 0.80 |  |
| 13,500 | 0.84 (.16) | 0.75 (.25) | 0.57 | 0.92 | 0.82 |  |
| 14,000 | 0.89 (.11) | 0.72 (.28) | 0.65 | 0.92 | 0.85 |  |
| 14,500 | 0.93 (.07) | 0.65 (.35) | 0.72 | 0.90 | 0.87 |  |

| Additional file 1: Table S12b. Uncensored steps/day for 60 MVPA minutes (Evenson) among girls 12-17 years old (n=656). | | | | | | |
| --- | --- | --- | --- | --- | --- | --- |
| Steps/day | Specificity (False Positive) | Sensitivity (False Negative) | PPV | NPV | Accuracy |  |
| 6,500 | 0.13 (.87) | 0.97 (.03) | 0.07 | 0.99 | 0.18 |  |
| 7,000 | 0.17 (.83) | 0.97 (.03) | 0.08 | 0.99 | 0.23 |  |
| 7,500 | 0.22 (.78) | 0.97 (.03) | 0.08 | 0.99 | 0.27 |  |
| 8,000 | 0.29 (.71) | 0.97 (.03) | 0.09 | 0.99 | 0.33 |  |
| 8,500 | 0.36 (.64) | 0.95 (.05) | 0.10 | 0.99 | 0.40 |  |
| 9,000 | 0.44 (.56) | 0.95 (.05) | 0.11 | 0.99 | 0.47 |  |
| 9,500 | 0.50 (.50) | 0.92 (.08) | 0.12 | 0.99 | 0.53 |  |
| 10,000 | 0.58 (.42) | 0.92 (.08) | 0.13 | 0.99 | 0.60 |  |
| 10,500 | 0.64 (.36) | 0.92 (.08) | 0.15 | 0.99 | 0.65 |  |
| 11,000 | 0.72 (.28) | 0.92 (.08) | 0.19 | 0.99 | 0.73 |  |
| 11,500 | 0.79 (.21) | 0.85 (.15) | 0.22 | 0.99 | 0.79 |  |
| 12,000 | 0.83 (.17) | 0.82 (.18) | 0.26 | 0.98 | 0.83 |  |
| 12,500 | 0.88 (.12) | 0.77 (.23) | 0.31 | 0.98 | 0.87 |  |
| 13,000 | 0.91 (.09) | 0.74 (.26) | 0.36 | 0.98 | 0.90 |  |
| 13,500 | 0.94 (.06) | 0.72 (.28) | 0.45 | 0.98 | 0.92 |  |
| 14,000 | 0.95 (.05) | 0.67 (.33) | 0.50 | 0.98 | 0.93 |  |
| 14,500 | 0.97 (.03) | 0.62 (.38) | 0.60 | 0.97 | 0.95 |  |

PPV=positive predictive value. NPV = negative predictive value. Accuracy = true positives + true negatives / (true positives + true negatives + false positives + false negatives).
